# Localization of an *hTERT *repressor region on human chromosome 3p21.3 using chromosome engineering

**DOI:** 10.1186/2041-9414-1-6

**Published:** 2010-05-26

**Authors:** Satoshi Abe, Hiromi Tanaka, Tomomi Notsu, Shin-ichi Horike, Chikako Fujisaki, Dong-Lai Qi, Takahito Ohhira, David Gilley, Mitsuo Oshimura, Hiroyuki Kugoh

**Affiliations:** 1 Department of Biomedical Science, Graduate School of Medical Science, and Chromosome Engineering Research Center, Tottori University, 86 Nishicho, Yonago 683-8503, Japan; 2 Department of Medical and Molecular Genetics, Indiana University School of Medicine, 975 West Walnut Street, IB-130, Indianapolis, IN 46202-5251, USA; 3 Division of Regenerative Medicine and Therapeutics, Graduate School of Medical Science, Tottori University, 86 Nishicho, Yonago 683-8503, Japan; 4 Frontier Science Organization, Institute for Gene Research, Kanazawa University, 13-1 Takaramachi, Kanazawa, 920-0934, Japan

## Abstract

Telomerase is a ribonucleoprotein enzyme that synthesizes telomeric DNA. The reactivation of telomerase activity by aberrant upregulation/expression of its catalytic subunit *hTERT *is a major pathway in human tumorigenesis. However, regulatory mechanisms that control *hTERT *expression are largely unknown. Previously, we and others have demonstrated that the introduction of human chromosome 3, *via *microcell-mediated chromosome transfer (MMCT), repressed transcription of the *hTERT *gene. These results suggested that human chromosome 3 contains a regulatory factor(s) involved in the repression of *hTERT*. To further localize this putative *hTERT *repressor(s), we have developed a unique experimental approach by introducing various truncated chromosome 3 regions produced by a novel chromosomal engineering technology into the renal cell carcinoma cell line (RCC23 cells). These cells autonomously express ectopic *hTERT (exohTERT) *promoted by a retroviral LTR promoter in order to permit cellular division after repression of endogenous *hTERT*. We found a telomerase repressor region located within a 7-Mb interval on chromosome 3p21.3. These results provide important information regarding *hTERT *regulation and a unique method to identify *hTERT *repressor elements.

## Background

Telomerase is a specialized reverse transcriptase that synthesizes telomeric DNA at the ends of most eukaryotic chromosomes. Human telomerase is composed of an RNA moiety (*hTR*) and a protein catalytic subunit (*hTERT*) [1]. In humans, telomerase activity is absent or greatly reduced in most somatic cells but present in germ and stem cells, and highly reactivated in the majority of human cancers [2,3]. Several reports suggest that *hTERT *protein levels reflect the amount of nuclear telomerase activity [4,5]. The *hTERT *gene appears to be controlled transcriptionally, though various post-transcriptional mechanisms have been suggested to be involved in *hTERT *regulation. Several lines of evidence suggest that the regulation of *hTERT *transcription involves both repressive and activating transcription mechanisms [6,7]. However, the *hTERT *transcriptional regulatory system is highly complex and largely unknown. Therefore, the identification of *hTERT *repressor(s) is an important step in understanding how telomerase is controlled during development, aging and tumorigenesis.

We previously reported that the introduction of a normal human chromosome 3 restored cellular senescence in two immortal renal cell carcinoma cell lines, RCC23 and KC12, using microcell-mediated chromosome transfer (MMCT) [8,9]. This inactivation of immortal growth by human chromosome 3 transfer was attributed to the loss of telomerase activity due to the transcriptional repression of the *hTERT *gene [9,10]. This work suggested that human chromosome 3 contains a locus responsible for *hTERT *repression.

Here we report a unique approach using MMCT to introduce truncated chromosome 3 regions into cells forcibly expressing *hTERT *to identify an *hTERT *repressor(s). By the transfer of each truncated human chromosome 3, we successfully identified a 7-Mb region within 3p21.3 containing an *hTERT *repressor(s). We present this approach as a useful tool for the identification and further analysis of *hTERT *repressor(s).

## Results

### Construction of donor cells with truncated human chromosome 3 fragments

**Figure 1:**
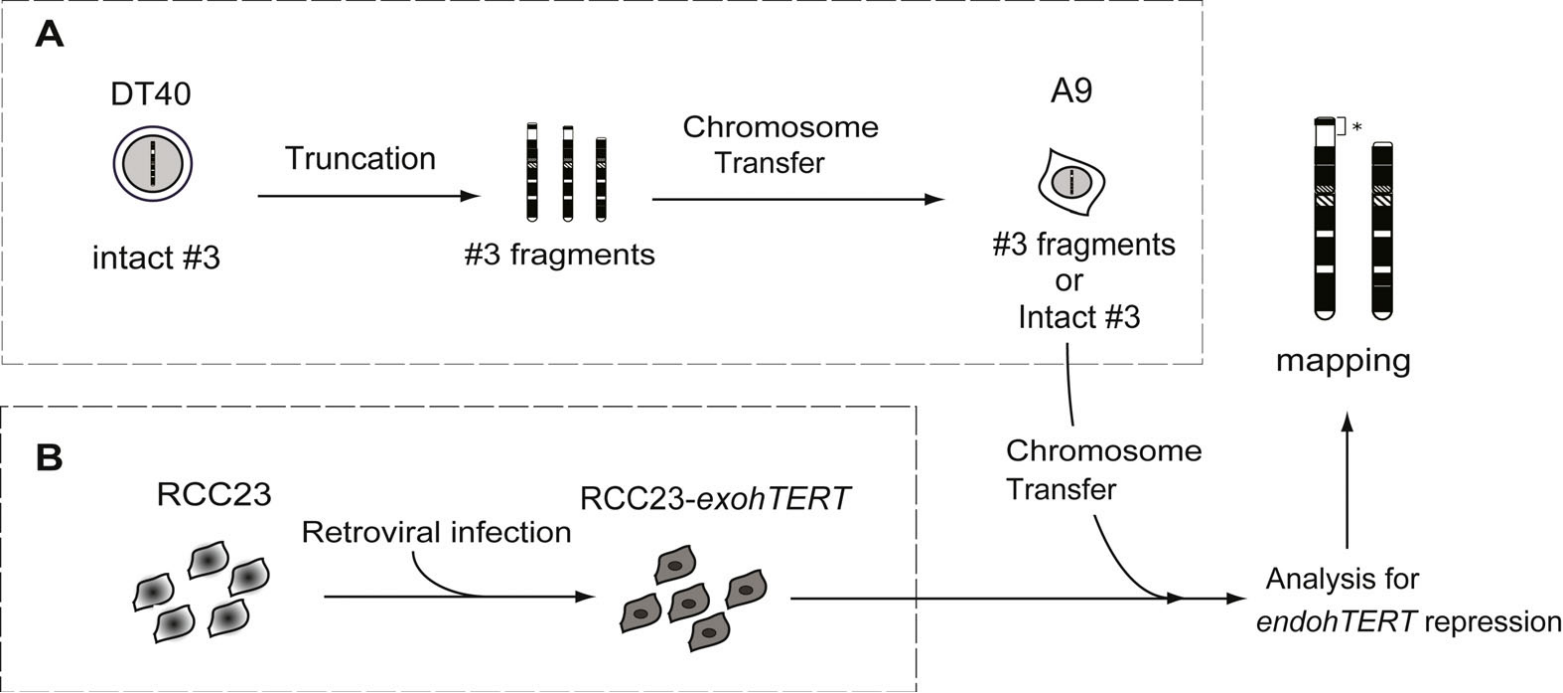
**The mapping approach for the identification of an *hTERT *repressor**. **(A) **Generation of truncated human chromosome 3 fragments was schematically shown. **(B) **Creation of RCC23 cells expressing ectopic *hTERT *by retroviral infection. An intact or truncated chromosome 3 (#3) fragments were transferred into RCC23-*exohTERT *cells. See text for details.

To determine genomic regions that contain *hTERT *repressor gene(s), we carried out MMCT of several truncated chromosome 3 into RCC23 renal cell carcinoma cells. The overall strategy for this approach is shown in Figure 1.

**Figure 2:**
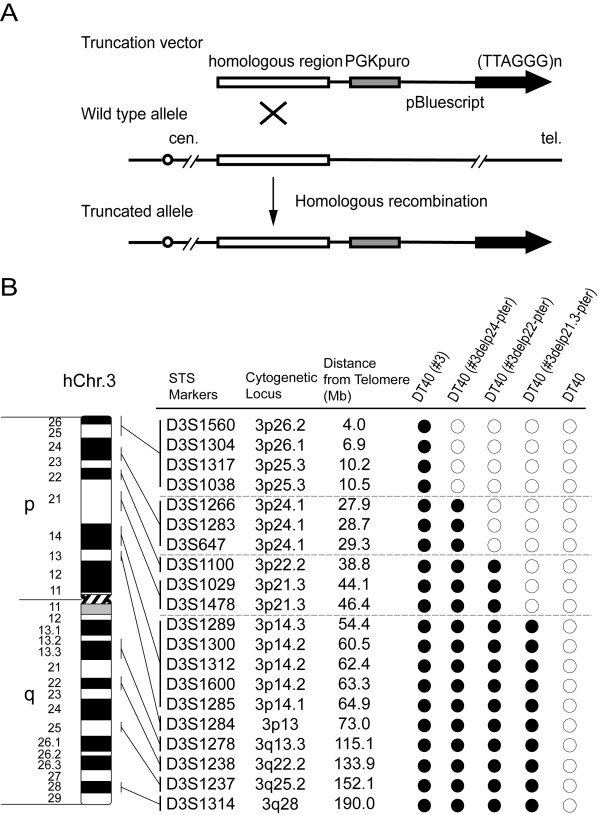
**Construction of truncated chromosome 3 in DT40 cells by telomere seeding**. **(A) **Targeting strategy of generating truncated chromosome. **(B) **Summary of PCR analyses on truncated chromosomes in DT40 cells. Twenty STS markers on chromosome 3 examined were shown. Solid circles and open circles represent presence and absence of truncated allele at tested loci in DT40 cells, respectively.

**Figure 3:**
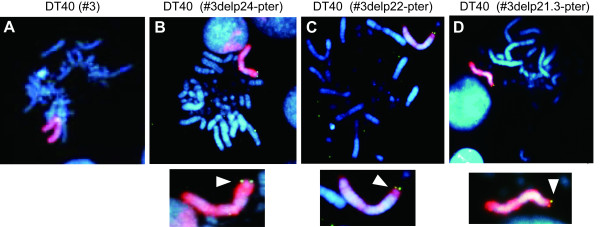
**FISH analysis of truncated human chromosome 3 in DT40 cells**. Upper panels show representative metaphase spreads (A-D) and bottom panels show the enlarge image of human chromosome with allow. The allow shows a targeting site on chromosome 3 using biotin-labeled PGK-puro probe (*green color*). **(A) **DT40 containing an intact human chromosome 3. **(B) **DT40 containing #3delp24-pter. **(C) **DT40 containing #3delp22-pter. **(D) **DT40 containing #3delp21.3-pter. Digoxigenin-labeled human COT-1 probe (*red color*) indicates human chromosome. DAPI staining (*blue color*) shows chicken chromosomes.

To successfully create various truncated chromosome 3 constructs, we developed a unique targeting vector. As shown in Figure 2A, there are three main components of this targeting construct vector: 1) ~1 kb of terminal telomeric repeats, 2) a puromycin resistant gene and, 3) ~4-10 kb of homologous sequences for targeting specific region of human chromosome 3. We used chicken DT40 pre-B-cells for targeted truncation of chromosome due to its high homologous recombination frequency. Initially, a neomycin-tagged intact human chromosome 3 (intact #3) was transferred into DT40 cells by MMCT (Figure 1A). Resistant DT40 microcell hybrid clones stably maintaining human chromosome 3 were isolated by G418 drug selection [termed DT40(#3)] (Figure 3A). Subsequently, we generated three targeting vectors using different homologous sequences; 1) 10 kb from 3p24 locus, 2) ~4 kb from 3 p22 locus, and 3) ~8 kb from 3p21.3 locus. After transfection of targeting vectors into DT40(#3) cells by electroporation, resistant clones were isolated by G418/puromycin double drug selection (Figure 1A). We confirmed the successful recombination of targeting vectors to human chromosome 3 by Southern blotting and PCR analysis using twenty Sequence-Tagged-Site (STS) markers located on the human chromosome 3 (Figure 2B). The order of STS markers and physical distance among them are based on a YAC contigs of WIGR database, UCSC Human Genome Browser database and human genome resource of the National Center for Biotechnology Information [11-13]. We isolated clones with truncated chromosome at 3p24 locus (DT40(#3delp24-pter)), 3p22 locus (DT40(#3delp22-pter)) and 3p21.3 locus (DT40(#3delp21.3-pter)) by FISH analysis. FISH analysis showed that all clones underwent targeted truncation at the three homologous regions (*green *dots in Figure 3B-C). Targeting ratio was 4 of 39 (10.36%) in DT40(#3delp24-pter) clone, 1 of 238 (0.42%) clone in DT40(#3delp22-pter) clone and 1 of 120 (0.83%) in DT40(#3delp21.3-pter) clone. It is likely that recombination efficiency not only depends on the length of homologous regions but also primary sequences within these targeting homologous regions.

To increase the efficiency of chromosome transfer into recipient cells, we transferred each truncated chromosome 3 into mouse A9 cells (Figure 1A). Following MMCT, we successfully isolated A9 microcell hybrid clones from each DT40(#3delp24-pter), DT40(#3delp22-pter) and DT40(#3delp21.3-pter) donor cells and each truncated human chromosome 3 was confirmed by FISH analysis to isolate clones containing a single truncated human chromosome [termed A9(#3delp24-pter), A9(#3delp22-pter) and A9(#3delp21.3-pter), respectively] (data not shown). Under drug selection in culture condition, the truncated human chromosome 3 was retained stably in mouse A9 cells (data not shown).

### Establishment of the RCC23 cells with autonomous ectopic *hTERT *expression and introduction of truncated chromosome 3 fragments

**Figure 4:**
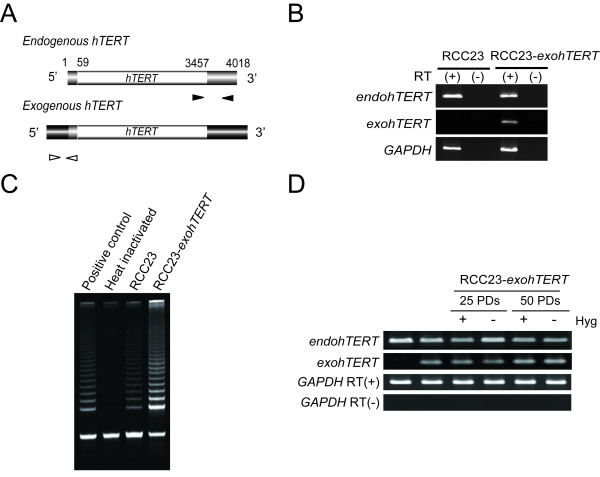
**Generation and characteristics of RCC23 cells expressing ectopic *hTERT *(A) **Distinct PCR primer design between endogenous and *exogenous hTERT *genes. Native UTR sequence was shown *in gray rectangle*. Additional sequences only in exogenous *hTERT *cDNA were shown *in dark rectangle*. Solid allow heads, primers for endogenous *hTERT*. Open allow heads, primers for endogenous *hTERT*. **(B) **Detection of ectopic *hTERT *expression in RCC23-*exohTERT *cells. RT-PCR was performed with (+) or without (-) reverse transcriptase (RT). *endohTERT*, endogenous *hTERT*. *exohTERT*, exogenous *hTERT*. **(C) **Upregulation of telomerase activity in RCC23-*exohTERT *cells. Enzymatic activity was heat-inactivated for a negative control. **(D) **Maintenance of *exohTERT *expression in RCC23-*exohTERT *cells during long-term culture. RCC23-*exohTERT *cells were cultured in the presence or absence of hygromycine B (Hyg, drug resistant marker of retroviral vector) until 25 PDs or 50 PDs and RT-PCR was performed for *hTERT *expression.

Previously, we found that the introduction of human chromosome 3 in RCC23 cells causes telomerase repression, along with the growth arrest after 10-30 population doublings (PDs) [10]. However, due to this severe growth arrest after telomerase repression, it was not possible to obtain sufficient cell numbers for functional studies. Interestingly, several reports suggest that a telomerase repressor located on human chromosome 3 may suppress E-box element mediated *hTERT *transcription [14-16]. Therefore we established an RCC23 cell line with autonomous expression of ectopic *hTERT *(*exohTERT*) with a retroviral LTR promoter (termed RCC23-*exohTERT*), in order to permit cellular division after chromosome 3 transfer (Figure 1B). An additional advantage of this retroviral vector is that *exohTERT *can be distinguished from endogenous *hTERT *by RT-PCR analysis using specific primer sets (Figure 4A, B). We confirmed that *exohTERT *was functional in RCC23-*exohTERT *cells by the increase of telomerase activity and the telomere elongation compared to RCC23 parental cell population (Figure 4C and data not shown). We also found that long-term culture (~50 PDs) did not change expression levels of both endogenous *hTERT *and *exohTERT *(Figure 4D).

**Figure 5:**
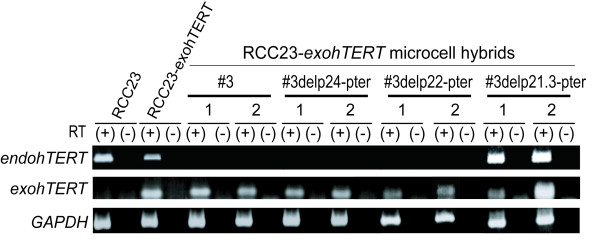
***hTERT *repression effect on #3delp24-pter and #3del22-pter, but not #3delp21.3-pter **Two clones of each RCC23-*exohTERT *microcell hybrids were examined by RT-PCR. *GAPDH *was used as an internal control. RT-PCR was performed with (+) or without (-) reverse transcriptase (RT). Note that the RCC23-*exohTERT *microcell hybrid containing an intact (#3) or the truncated human chromosome 3, #3delp24-pter or #3delp22-pter has no expression of endogenous *hTERT *gene (*endohTERT*).

Using the RCC23-*exohTERT *cells, we first introduced an intact human chromosome 3 *via *MMCT. As expected, transferred chromosome 3 in RCC23-*exohTERT *cells suppressed endogenous *hTERT *expression, nevertheless the cells expressed *exohTERT *and continued to grow normally without morphologic change (Figure 5). This result suggests that ectopic *exohTERT *expression permits telomerase activity with endogenous *hTERT *repression, thereby preventing cellular senescence in these microcell hybrid cells. To explore which truncated chromosome fragment contains *hTERT *repression, three truncated chromosomes were transferred into RCC23-exo*hTERT *cells. We isolated three microcell hybrid clones with truncated chromosome (#3delp24-pter and #3delp21.3-pter) and nine microcell hybrid clones with truncated chromosome (#3delp22-pter) and examined the expression profiles of *hTERT *in each microcell hybrid clones by RT-PCR analysis (Figure 5). Microcell hybrids generated by the introduction of truncated human chromosome 3, all RCC23-*exohTERT*(#3delp24-pter) and RCC23-*exohTERT*(#3delp22-pter) clones, showed complete repression of endogenous *hTERT *expression. Conversely, the remaining RCC23-*exohTERT*(#3delp21.3-pter) microcell hybrid clone had no effect. These results indicate the presence of the *hTERT *repressor locus on the 3p21.3-p22 region commonly retained in truncated chromosomes #3delp24-pter and #3delp22-pter, but not #3delp21.3-pter.

### Chromosome mapping for the identification of region containing the *hTERT *repression effect

**Figure 6:**
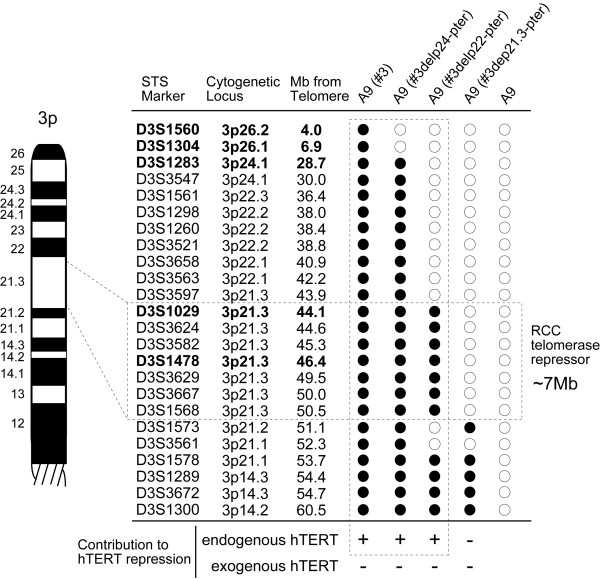
**Identification of an *hTERT *repressor locus on human chromosome 3p21.3 **Twenty-four STS markers on 3p examined were shown. *Open circles*, absence of tested loci in transferred human chromosome. *Closed circles*, presence of tested loci in transferred human chromosome. STS markers *in bold *were used in Figure 2b as well. Note that 7-Mb interval between D3S3597 and D3S1573 on 3p21.3 contains the effect of endogenous *hTERT *repression into RCC23-*exohTERT *cells. Interstitial deletion of transferred chromosome was observed on D3S1573 and D3S3561 loci in A9(#3delp2-pter) clone, and D3S1573 locus in A9(#3del21.3-pter) clone.

To determine which region in 3p21.3-p22 carries the telomerase repressor element, we performed PCR analysis of A9 microcell hybrid clones using a total of 24 STS markers located on the human chromosome 3 (Figure 1 and 6). We found that seven STS markers (from D3S1029 to D3S1568) were present commonly in an intact chromosome 3, two truncated chromosomes #3delp24-pter and #3delp22-pter which had *hTERT *repression effect (Figure 5 and 6). However truncated chromosome #3delp21.3-pter which had no effect on the *hTERT *expression lost these seven markers (Figure 5 and 6). Thus, these results indicate that 7-Mb interval between D3S3597 and D3S1573 on 3p21.3 contains the region that significantly affect repression of *hTERT *expression, suggesting that this minimal region controls telomerase activity through suppression of *hTERT *expression in RCC23 cells.

## Discussion

We have previously shown that the introduction of human chromosome 3 suppresses telomerase activity due to the repression of the *hTERT *transcription. Here, we report that the result of the fine mapping of a telomerase repressor gene within the human chromosome 3p21.3 region by functional analysis of various truncated chromosome 3 fragments.

To further isolate the telomerase repressor region(s) on chromosome 3, we utilized MMCT using several truncated chromosome 3 in RCC23-*exohTERT *cells. Unlike X-irradiated chromosome transfer method [17] or deletion mapping [18], this targeted truncation technique allows the removal of specific regions on a targeted chromosome using homologous DNA sequences. Additionally, chicken DT40 cells are able to facilitate truncation efficiency of chromosome at desired sites [19-21]. Indeed, we could truncate chromosome with the high targeting efficiency at least forty-fold using DT40 cells compared to <0.01% with conventional methods [22].

Gene functional analysis by MMCT from mouse A9/human monochromosomal hybrids to suitable recipient cells, has been utilize to map responsible genes involved in cellular aging, metastasis, DNA repair and tumor suppression [23]. Because the repression of *hTERT *transcription results in the induction of cellular senescence it was not possible to obtain sufficient cell numbers for functional studies. In this study, RCC23-*exohTERT *cells ectopically expressing *exohTERT *with a viral LTR promoter enable us to study endogenous *hTERT *repression by preventing cellular senescence. Thus, RCC23-*exohTERT *cells will serve as a valuable resource for the mapping and identification of genes that function during cellular aging involving telomerase regulation.

Using MMCT, we have shown that the introduction of either chromosome 3 or 10 and truncated chromosome 3 results in the complete repression of *hTERT *transcription in human tumor cells [8,24], suggesting that there are multiple telomerase-dependent pathways for the regulation of cellular senescence [25]. However, human/mouse A9 monochromosomal hybrids that carry these human chromosomes used as donor were all positive for telomerase activity and expressed *mtert*. It is likely that this phenomenon was caused by some significant differences between human and mouse biology. Specifically, telomeres in inbred mouse strains are on average much longer than those in humans. Moreover, the expression of *hTERT *is more tightly regulated than that of *mtert *in normal somatic cells, although the expression of both genes is strongly up-regulated in mouse or human tumors.

Tanaka et al. (2005) previously reported that a renal cell carcinoma cell line, KC12 shared the same genetic defect of telomerase repressor function as the RCC23 cell line using a genetic complementation approach [26]. Remarkably, the candidate region that was found using deletion mapping of KC12 revertant clones that escaped from cellular senescence overlapped with the region identified in this report [26]. When we identified the telomerase repressor gene(s) within this candidate region using the functional analysis of truncated chromosomal regions within this study, it confirmed the effect of *hTERT *suppression found in KC12 cells. In addition, we did not check the effect of chromosome 3 for expression of *hTERT *in other type of human cells. However, it has been reported that genomic abnormalities in the chromosome 3p and 3p21 region are present in various types of human cancers [27]. Therefore, identification of a telomerase repressor gene(s) within this region should facilitate our understanding of the molecular mechanisms that are involved in cancer development.

Consistent with previous reports, we identified a candidate region that contains a telomerase repressor element on chromosome 3p21.3 within a 7-Mb region [26]. Conversely, Szutorisz et al. (2003) mapped another *hTERT *repressor gene of a breast carcinoma cell line 21NT at a distinct locus on chromosome 3p [28]. Taken together, we have evidence that renal cell carcinoma (RCC23 and KC12) and breast carcinoma (21NT) have different defects in telomerase repression. This implicates that there are at least two telomerase repressors on human chromosome 3p and the tissue-specific regulation of telomerase might be involved during human development and carcinogenesis [26].

Renal cell carcinoma shows frequent deletion of the short-arm of human chromosome 3 [27,29]. The candidate region on 3p21.3 harbors various tumor suppressor genes (*HYAL2, FUS1, RASSF1, BLU, NPR2L, CYB561D2, PL6 *and *CACNA2D2*). It is unlikely that RASSF1A is an *hTERT *repressor gene on chromosome 3p21.3 because the forced expression of RASSF1 in RCC23 cells did not repress *hTERT *promoter activity [26]. Other candidate genes on 3p21.3 will need to be analyzed. Additionally, novel microRNAs on 3p21.3 may potentially affect transcriptional regulation of *hTERT*, since numerous microRNAs have recently been shown as regulatory factors for tumor suppression [30]. In fact, it was reported that overexpression of miR-138 (mapped to 3p21) downregulated hTERT protein level but not *hTERT *mRNA in human thyroid carcinoma cell lines. It is thus unlikely that the miR-138 functions as a transcriptional repressor of *hTERT *[31]. Taken together, human chromosome 3 may harbor multiple telomerase regulating factors that act in a tissue-specific manner or through different pathways such as telomerase inactivation with or without *hTERT *transcriptional repression.

The combined use of RCC23-*exohTERT *cells and chromosomal engineering technique is expected to facilitate the positional cloning of the telomerase repressor by a series of cutting down the 7-Mb region and trap the telomerase repressor locus in YAC (Yeast Artificial Chromosome) or BAC/PAC (Bacterial Artificial Chromosome/P1-derived Artificial Chromosome) clones. Furthermore, our group previously reported the construction of a human artificial chromosome (HAC) vector that has the capability to clone extremely large genomic regions and a utility for potential gene therapeutic uses [32]. The HAC vector is maintained independently in cells without genomic integration because it contains autonomous elements such as telomeres, centromeres and replication origins [33]. The combination of the HAC vector and human genome BAC/PAC libraries will be a useful functional analysis approach to evaluate a cluster of genes [34]. Additionally, positional cloning strategies such as the TAR cloning method allows direct cloning of a specific chromosomal region from the human genome using *in vivo *recombination in yeast [35,36]. The precise mapping information of candidate genes to a defined region of a human chromosome will provide the necessary information for isolation of telomerase repressor genes and help lead to mechanistic understanding of the control of *hTERT *transcription.

## Materials and methods

### Cell culture

Chicken DT40 cells containing chromosome 3 were maintained in RPMI-1640 (Invitrogen, Carlsbad, CA) with 10% fetal bovine serum (JRH Biosciences, Lenexa, KS), 1% chicken serum (Invitrogen), 50 μM 2-mercaptoethanol (Sigma), 1.5 mg/ml G418 (Invitrogen) and the appropriate antibiotics. DT40 hybrid cells containing truncated chromosomes were selected with 1.5 mg/ml G418 and 0.3 μg/ml puromycin (Sigma). A9 hybrid cells carrying intact or truncated chromosomes were selected with 800 μg/ml G418. Human renal cell carcinoma cell line RCC23 was maintained in RPMI-1640 supplemented with 10% FBS.

### Microcell-mediated chromosome transfer

MMCT was performed to introduce truncated human chromosomes from DT40 to A9 cells as described previously [33]. Briefly, 1 × 10^9 ^microcells were prepared by centrifuge in coating flasks (Nalge Nunc, Rochester, NY) with poly-L-lysine (Sigma) and were fused to A9 cells by 47% polyethylene glycol 1000 (WAKO, Japan). Chromosome transfer into RCC23-*exohTERT *cells was performed using standard procedures [37]. A9 hybrid clones were treated with 0.05 μg/ml of colcemid to induce the formation of micronuclei, which were then purified by 10 μg/ml cytochalasin B (Sigma) digestion and centrifugation. After centrifugation, the isolated microcells were resuspended in serum-free DMEM and filtered sequentially through 8-, 5-, and 3-μm polycarbonate filter (Whatman). The purified microcells were collected by centrifugation at 400 × *g *for 15 min and resuspended in serum-free DMEM containing 50 μg/ml of phytohemagglutinin-P (Wako). The microcells were attached to monolayer cells at 37°C for 15 min. The microcells were fused with recipient cells in a 47% PEG solution for 1 min, followed by extensive washing with serum-free DMEM. The cells were maintained in nonselective medium for 24 hr, then trypsinized and split into three 100-mm dishes containing selection medium.

### Generation of DT40 cells with truncated human chromosome 3

For chromosome truncation at 3p24 locus, cosmid cCI3-1202 was obtained from Japanese Collection of Research Bioresources (JCRB) for construction of a targeting vector. 10 kb of *Eco*RI-digested DNA form cCI3-1202 was used as a homologous sequence. For chromosome truncation at 3p22 and 3p21.3 loci, cosminds c305 and Z84494 were used as a PCR template to amplify the homologous region. The following primer sets were used for PCR; 5'-ATG GAT CCA ACA CAC ACC ACC CTC ATG TAT GTC CA-3' and 5'-ATG GAT CCC AGA CCA AGT CCG GAA GAG CTG CTT-3' for 3p22 homologous region, 5'-ATC CCT GGC CTG TCA CCA CT-3' and 5'-GGC CTG ACA GCA GCA CAT TT-3' for 3p21.3 homologous region. The PCR product was cloned into *Bam*HI-digested pBS-TEL/Puro vector [22] by ligation. Targeting constructs which contain telomeric repeats, puromycin resistant gene and homologous sequence were linearized and transfected by electroporation under condition at 550 V and 25 μF into DT40 cells carrying a human chromosome 3 as described previously [33]. The cells were resuspended in basic growth medium and aliquoted into four 96-well plates. After 2 days, the cells were resuspended in selective medium with puromycin. After 2 week, drug resistant colonies were isolated and expanded for subsequent analyses. The recombination was confirmed by Southern blotting or PCR.

### Generation of RCC23 cells expressing ectopic hTERT

Retroviral protein expression vector, pBABE(hyg)-*hTERT *was used for introduction of ectopic *hTERT *gene (*exohTERT*) into RCC23 cells. The construct is a kind gift from Dr. Collins (UC Berkeley, USA) [38]. Viral supernatant was generated by stable transfection of a packaging cell line (PT67, Clontech). RCC23 cells were seeded at 2 × 10^5 ^cells per 60 mm dish at 1 day before infection. For infection, the culture medium was replaced by viral supernatant everyday for three days. After overnight of the third infection, the infected cells were selected by culture in 200 μg/ml hygromycin B.

### RNA isolation and RT-PCR for hTERT expression

Total RNA was extracted by using RNeasy mini kit (Qiagen) and treated with DNase I (Wako). The first-strand cDNA was synthesized using M-MLV reverse transcriptase (Invitrogen) with the oligo(dT)_15 _primer. cDNA was amplified using 5'-CGA GAG CAG ACA CCA GCA G-3' and 5'-TTTTACTCCCACAGCACCTC-3' for endogenous *hTERT*; 5'-GAC GAC GAT GAC AAG GGA AT-3' and 5'-AGC ACC TCG CGG TAG TGG-3' for exogenous *hTERT*; 5'-CCA TCT TCC AGG AGC GAG A-3' and 5'-TGT CAT ACC AGG AAA TGA GC-3' for *GAPDH*. The PCR reaction for endogenous *hTERT *was performed using Advantage2 PCR kit (Clontech).

### Genomic PCR analysis

The presence or absence of the region on human chromosome 3 was checked by PCR using 24 specific sequence-tagged site (STS) markers (D3S1560, D3S1304, D3S1317, D3S1038, D3S1266, D3S1283, D3S647, D3S1100, D3S1029, D3S1478, D3S1289, D3S1300, D3S1312, D3S1600, D3S1285, D3S1284, D3S1278, D3S1238, D3S1237, D3S1314, D3S3547, D3S1561, D3S1298, D3S1260, D3S3521, D3S3658, D3S3563, D3S3597, D3S3624, D3S3582, D3S3629, D3S3667, D3S1568, D3S1573, D3S3561, D3S1578, D3S3672). Primer information was obtained from NCBI database [11]. PCR was processed through 35 cycles of PCR consisting of 30 sec at 94°C, 30 sec at 58-62°C and 30 sec at 72°C.

### FISH analysis

FISH analysis was performed using fixed metaphase spreads of each DT40 cells carrying intact or truncated chromosomes and the combination with digoxigenin-labeled (Roche, Basel, Switzerland) human COT-1 DNA (Invitrogen) and biotin-labeled PGK-puro plasmid DNA as described previously [39]. For metaphase of each A9 hybrid carrying intact or truncated chromosomes, digoxigenin-labeled human COT-1 DNA was used for the detection of the human chromosome. Chromosomal DNA was counterstained with DAPI (Sigma). The images were captured using the Argus system (Hamamatsu Photonics, Hamamatsu, Japan).

### Telomerase activity assay

Telomerase activity was measured by TeloChaser (TOYOBO, Japan) according to the manufacturer's protocol. The reaction products were electrophoresed on a 12.5% nondenaturing polyacrylamide gel and visualized by SYBR GREEN I staining (Invitrogen).

## Competing interests

The authors declare that they have no competing interests.

## Authors' contributions

SA carried out molecular genetic study and drafted the manuscript. HT participated in the design of the study and performed molecular genetic study. TN carried out the construction of vectors. SH carried out chromosome engineering. DQ carried out FISH analysis. TK carried out examination of chromosome transfer. DG participated in the design of the study and coordination and helped to draft manuscript. MO helped to draft manuscript and coordination. HK conceived of the study, and participated in the design of the study and coordination and helped to draft manuscript. All authors read and approved the final manuscript.
